# Suppressive Effect of CORM-2 on LPS-Induced Platelet Activation by Glycoprotein Mediated HS1 Phosphorylation Interference

**DOI:** 10.1371/journal.pone.0083112

**Published:** 2013-12-20

**Authors:** Dadong Liu, Feng Liang, Xu Wang, Jie Cao, Weiting Qin, Bingwei Sun

**Affiliations:** Department of Burn and Plastic Surgery, Affiliated Hospital, Jiangsu University, Zhenjiang, China; University of Western Ontario, Canada

## Abstract

In recent years, it has been discovered that septic patients display coagulation abnormalities. Platelets play a major role in the coagulation system. Studies have confirmed that carbon monoxide (CO) has important cytoprotective and anti-inflammatory function. However, whether CO could alter abnormal activation of platelets and coagulation and thereby reduce the incidence of mortality during sepsis has not been defined. In this report, we have used CO-releasing molecules (CORM-2) to determine whether CO inhibits LPS-induced abnormal activation of platelets and have explored the potential mechanisms. LPS was used to induce activation of platelets *in vitro,* which were purified from the peripheral venous blood of healthy adult donors. CORM-2 was applied as a potential therapeutic agent. CORM-2 preconditioning and delayed treatment were also studied. We found that in the LPS groups, the function of platelets such as spreading, aggregation, and release were enhanced abnormally. By contrast, the platelets in the CORM-2 group were gently activated. Further studies showed that the expression of platelet membrane glycoproteins increased in the LPS group. Coincidently, both hematopoietic lineage cell-specific protein 1 and its phosphorylated form also increased dramatically. These phenomena were less dramatically seen in the CORM-2 groups. Taken together, we conclude that during LPS stimulation, platelets were abnormally activated, and this functional state may be associated with the signal that is transmitted between membrane glycoproteins and HS1. CORM-released CO suppresses the abnormal activation of platelets by interfering with glycoprotein-mediated HS1 phosphorylation.

## Introduction

Sepsis is a systemic inflammatory response syndrome caused by a severe systemic infection, and continues to be the leading cause of morbidity and mortality in severe hemorrhage, trauma, burns, or abdominal surgery patients, and accounts for approximately 90, 000 deaths each year in the United States [1]–[3]. The fundamental mechanism responsible for sepsis remains unknown, but it is associated bacterial infection, the release of inflammatory cytokine and coagulation abnormalities [4]. Recently, much evidence has demonstrated that disorders of the circulatory system plays a major role in sepsis [5], [6]. It is thought that sepsis is characterized by a number of coagulation disorders, including disseminated intravascular coagulation (DIC) [7], hemodynamic changes [8] and decreased systemic vascular resistance [3].

It is well known that platelet activation is an important event in activation of the coagulation system. Emerging evidence suggests that platelets may also play a critical role in many diseases involving the host immune response [9], inflammatory response [10], carcinogenesis [11] and so on. During sepsis, lipopolysaccharides (LPS, or endotoxin), and inflammatory cytokines (e.g., TNF-α) promote platelet activation, which then contributes to microthrombi formation in the capillaries [3], [12]. Meanwhile, activated platelets release or produce major functional proteins, some of which regulate inflammation and affect host immune function, such as interleukin (IL) 1-β, monocyte chemoattractant factor (MCP-1), and platelet factor 4 (PF4) [9], [13]. It is notable that both platelet membrane glycoproteins (GPIbα, GPVI) and HS1, which is a signaling molecule that functions downstream of glycoprotein activation, may contribute to platelet activation [14]–[16]. Thus, these studies provide novel insights of the potential clinical utility of anti-platelet therapy in the treatment of sepsis.

CO, commonly viewed as a silent killer, is a colorless, tasteless, and odorless gas. However, small amounts of CO are continuously produced in mammals, and the intracellular levels of this gaseous molecule can markedly increase under stressful conditions [8], [17]. In addition, some experiments have determined that the administration of exogenous CO exhibited important cytoprotective functions, and anti-inflammatory properties [6], [18]–[20]. However, as CO is known to be toxic at high concentration, the secure and optimal delivery of gaseous CO needs to be carefully conducted, and is even considered difficult.

Recently, transition metal carbonyls have been identified as potential CO-releasing molecules (CORMs) with the potential to facilitate the pharmaceutical use of CO by delivering it to the tissues and organs of interest [8], [21]. Studies elucidated that CORM-2 suppresses LPS-induced inflammatory responses in human umbilical vein endothelial cells (HUVECs), peripheral blood mononuclear cells (PBMCs) and macrophages [22], [23]. Similarly, many results have confirmed that CO derived from CORMs rescues mice from lethal endotoxemia and sepsis induced by LPS or cecal ligation and puncture (CLP) models [24]–[28].

Our previous studies have shown that CORM-2 inhibited over-expression of adhesion molecules, attenuated leukocyte sequestration in the organs of CLP or burn-induced septic mice, decreased intracellular oxidative stress and NO production in LPS-stimulated HUVECs [29]–[32]. However, no studies have previously assessed the effects of CORMs in regulating activation of the coagulation system, and interactions between inflammation and coagulation in sepsis. With the understanding of CORMs, we hypothesized that CORMs regulate platelet activity in the coagulation system during sepsis. In this study we demonstrated that membrane glycoproteins and HS1 play important roles in LPS-induced platelet activation. More significantly, our studies revealed that the molecular mechanisms involved in anticoagulant treatment might involve glycoprotein-mediated HS1 phosphorylation.

## Materials and Methods

### Ethics Statement

The Medical Ethical Committee of Jiangsu University approved the study. After written informed consent, blood specimens were obtained from the cubital veins of healthy donors. The Medical Ethical Committee of Jiangsu University gave consent for the use of these samples.

### Materials

CORM-2, dimethyl sulfoxide (DMSO), tyrodes solution, protease inhibitor cocktail, LPS and RIPA buffer, the FITC-labeled phalloidin and fibrinogen were obtained from Sigma-Aldrich (St. Louis, MO, USA). CORM-2 was solubilized in DMSO to obtain a 40 mmol/L stock. The inactive form of CORM-2 (negative control) was prepared as described previously [33]. FITC-labeled CD41 monoclonal antibody (mAb), eFluor 660-labeled GPVI mAb, and eFluor 660-labeled IgG1 were obtained from eBioscience (San Diego, CA, USA). Phosphatase inhibitor cocktail, actin goat polyclonal IgG, PE-labeled GPIbα mAb and PE-labeled IgG1 were obtained from Santa Cruz Biotechnology (Dallas, Texas, USA). HS1 (D83A8) XP(TM) rabbit mAb, Phospho-HS1 (Tyr397) antibody and anti-rabbit IgG HRP-linked antibody were obtained from Cell Signaling Technology (Boston, MA, USA). A FACSCalibur BD Flow Cytometer was obtained from Becton Dickison Corporation (San Jose, CA, USA). Incubator-CO_2_ (Napco 5400), high-speed cryogenic desktop centrifuges, and an automatic coagulation analyzer were obtained from Beckman Coulter Corporation (Pasadena, CA, USA). Image-Pro plus 6.0 software were obtained from Media Cybernetics (Maryland, USA).

### Preparation of Washed Platelets

Blood was extracted from healthy volunteers, after informed consent, and in accordance with the Medical Ethical Committee of Jiangsu University. Blood was collected into vacuum tubes and anti-coagulated with one-nine of 129 mM trisodium citrate. Platelet rich plasma (PRP) was obtained by centrifuging at 120×g for 10 min. Platelets were isolated by centrifuging at 678×g for 10 min and then washed twice with CGS buffer. The platelet poor plasma (PPP) was used to measure platelet aggregation. The platelets were resuspended in Tyrode’s buffer for at least 1 h at 37°C before use [34]. For experiments with PRP, the platelet density was maintained at 2×10^8^/mL for every experiment.

### Platelets Stimulation Model

LPS (10 µg/ml) was used to stimulate PRP to imitate the condition of coagulation under sepsis. This final concentration of LPS was used according to the published results in our laboratory and others [30], [35]. The PRP was assigned to five groups randomly. The control group did not undergo any treatment, whereas the LPS group received LPS simulation for 30 min, the CORM-2 group and iCORM-2 group underwent the same simulation and immediate administration of CORM-2 or iCORM-2 with different doses (10 and 50 µM), respectively. In addition, CORM-2 pre-conditioning and delayed treatment were also investigated. Briefly, CORM-2 preconditioning for 30 min followed by LPS stimulation for an additional 30 min was used as an induced platelet-preconditioning model; LPS stimulation for 30 min followed by CORM-2 treatment for an additional 30 min was used as an induced platelet delayed treatment model. Samples were incubated in a CO_2_-incubator at 37°C, 95% humidity, and 5% CO_2_. After the intervention, the correlation indices were detected.

### Platelet Adhesion and Spreading

Platelet spreading on immobilized fibrinogen was performed as previously described [36]–[38]. Fibrinogen was coated on the slides with microtiter wells at 4°C overnight. Platelet stimulation and CORM-2 intervention were performed as described above. Platelet adhering and spreading on fibrinogen-coated wells was performed at 37°C for 90 min. Then the cells were washed, fixed, permeabilized, and stained with FITC-labeled phalloidin. Adherent platelets were measured under a fluorescence microscope using a 40×ocular lens. The spreading area of platelets was measured using Image-Pro plus 6.0 software. Three randomly selected fields of view from different tests were used for statistical analysis.

### Platelet Aggregation

Platelet aggregation was measured in a platelet aggregometer as previously described [39]. Samples (300 µL) were incubated with magnetic bars at 37°C with stirring. ADP was applied to induce platelet aggregation. Data were recorded for 5 min.

### Platelet Secretion

Platelet secretion was determined by measuring adenosine triphosphate (ATP) release [16]. The effect of ATP release was examined using a luminometer. PRP were incubated with LPS at 37°C and at different time-points, ATP in the supernatant was measured by the addition of luciferin-luciferase reagent [35]. At the same time, ATP in the platelets was examined. Washed human platelets were lyzed in 200 µL of lysis buffer. Then 50 µL of the lyzed platelet solution and 50 µL of luciferin–luciferase reagent were mixed for 3 s before luminescence was measured for 10 s using a luminometer. Data were expressed as relative ATP levels [16], [40].

### Flow Cytometry

Samples of all groups were collected and fixed in 1% paraformaldehyde for 15 min at room temperature. The fixed samples were incubated with FITC-labeled CD41 (CD41-FITC). Then GPIV-eFluor 660 and GPIbα-PE were added into the above samples, independently. IgG1- eFluor 660 and IgG1-PE were applied as isotype control antibodies. All samples were incubated in the dark for 30 min at room temperature. Samples were washed three times and then analyzed by flow cytometry [41], [42].

### Immunoprecipitation and Western Blot

Platelet stimulation and CORM-2 intervention were performed as described above. The reaction was stopped by the addition of equal volumes of the RIPA buffer that contained protease and phosphatase inhibitor cocktails. The lysates were incubated for 30 min on ice for completion of the lysis action.

For immunoprecipitation studies, lysates were cleared by centrifugation and precleared for non-specific binding by rotating with beads alone before primary antibody against HS-1 (Cell Signaling) was added to the cleared lysates, and then rotated overnight at 4°C. TrueBlot anti-rabbit IgG beads (eBioscience) or Protein G Dynabeads (Invitrogen) were added to the lysates, and then the immunoprecipitates were washed and solubilized with 2×SDS buffer, boiled and loaded on 8% SDS–PAGE gels and processed as described below.

Samples (10 µg of protein) were subjected to electrophoresis on 8% SDS-polyacrylamide gels, with the use of the discontinuous system and transferred onto nitrocellulose membranes. The membranes were probed with anti-HS1 monoclonal antibody (1∶1000) or anti-Phospho-HS1 (Tyr397) followed by anti-rabbit IgG conjugated to horseradish peroxidase (1∶5000) as a secondary antibody. The bands were visualized using ECL reagent and Hyperfilm ECL (Amersham, Arlington Heights, IL, USA) as described by the manufacturer. Films were scanned using a flatbed scanner and the bands were quantified using Basic Quantifier software (Bio Image, Ann Arbor, MI, USA), which is an image analysis program [16].

### Statistical Analyses

Our data is presented as the mean ± standard deviation. Statistical analysis was performed by one-way ANOVA and the significant differences between two groups were analyzed by post-hoc test (SNK). An alpha value of P<0.05 was considered statistically significant.

## Results

### Effects of CORM-2 on Platelet Spreading in LPS-stimulated Platelets

**Figure 1:**
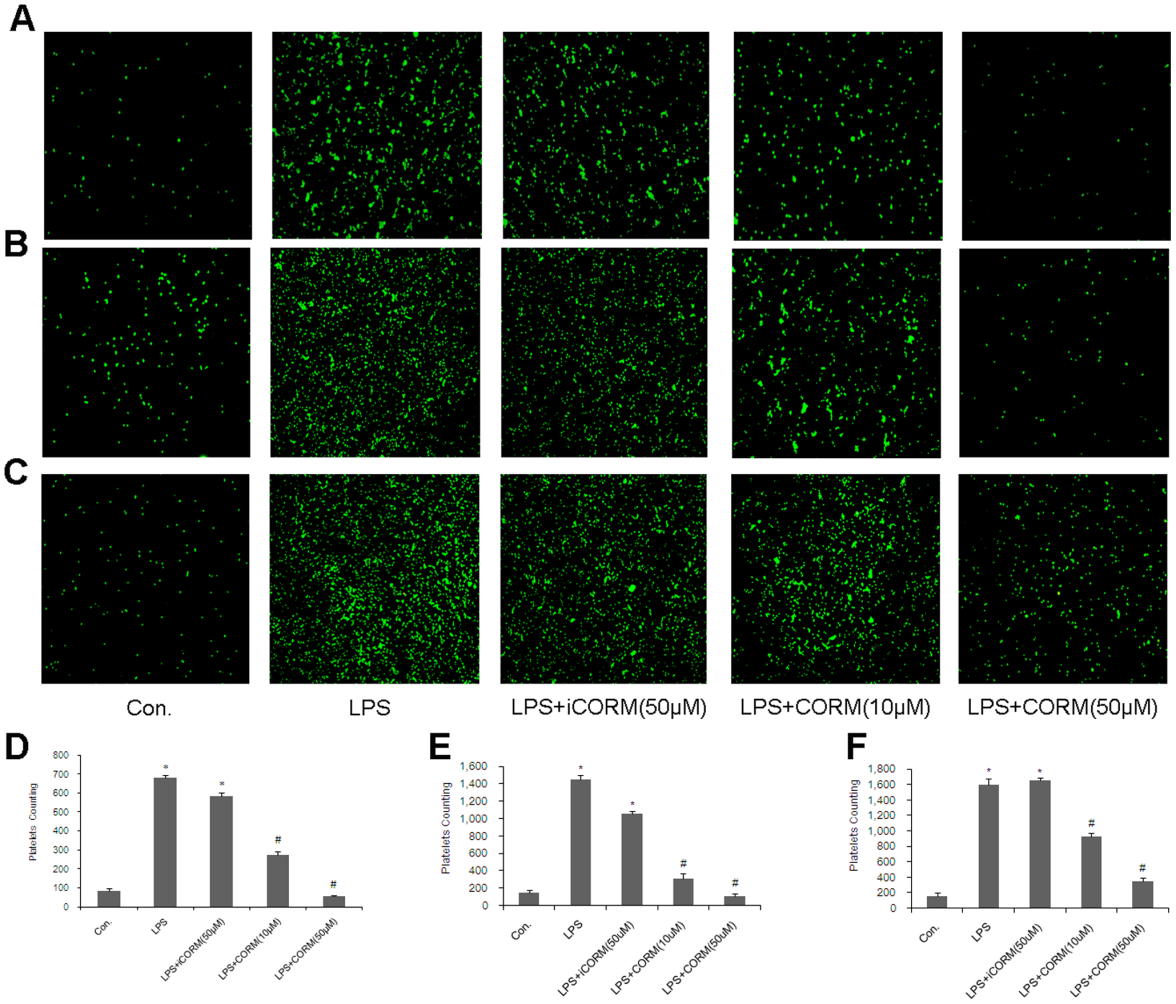
Effects of CORM-2 on platelet spreading following LPS stimulation. Platelets were stimulated by LPS (10 µg/ml) for 30 min in the presence or absence of CORM-2 (10, 50 µM). In addition, CORM-2 preconditioning and delayed treatment were also investigated as described in “Platelet stimulation model” section above. LPS stimulation significantly increased the spreading of platelet numbers as compared to the control groups, and treatment of platelets with CORM-2 (10, 50 µM) for 30 min significantly reduced platelet spreading in response to LPS stimulation (A, D). Similar results were also shown in CORM-2 pre-conditioning (B, E) and CORM-2 delayed treatment groups (C, F). Results are presented as mean ± SE of five experiments, *P<0.01 as compared to control; #P<0.05 as compared to LPS. Note that LPS-induced platelet spreading was inhibited by CORM-2 in a dose-dependent manner.

Platelet adhesion and spreading on fibrinogen are important indicators of thrombotic diseases. We found that LPS stimulation significantly increased the spreading of platelet numbers as compared to control groups, and treatment of platelet with CORM-2 (10, 50 µM) for 30 min significantly reduced platelet adhesion in response to LPS stimulation (Figure 1A, D). Interestingly, similar results were also shown in CORM-2 pre-conditioning (Figure 1B, E) and CORM-2 delayed treatment groups (Figure 1C, F). These results indicated that LPS can enhance platelet adhesion rate and exogenous CO can significantly reduce this increase.

### Effects of CORM-2 on Platelet Aggregation in LPS-stimulated Platelets

**Figure 2:**
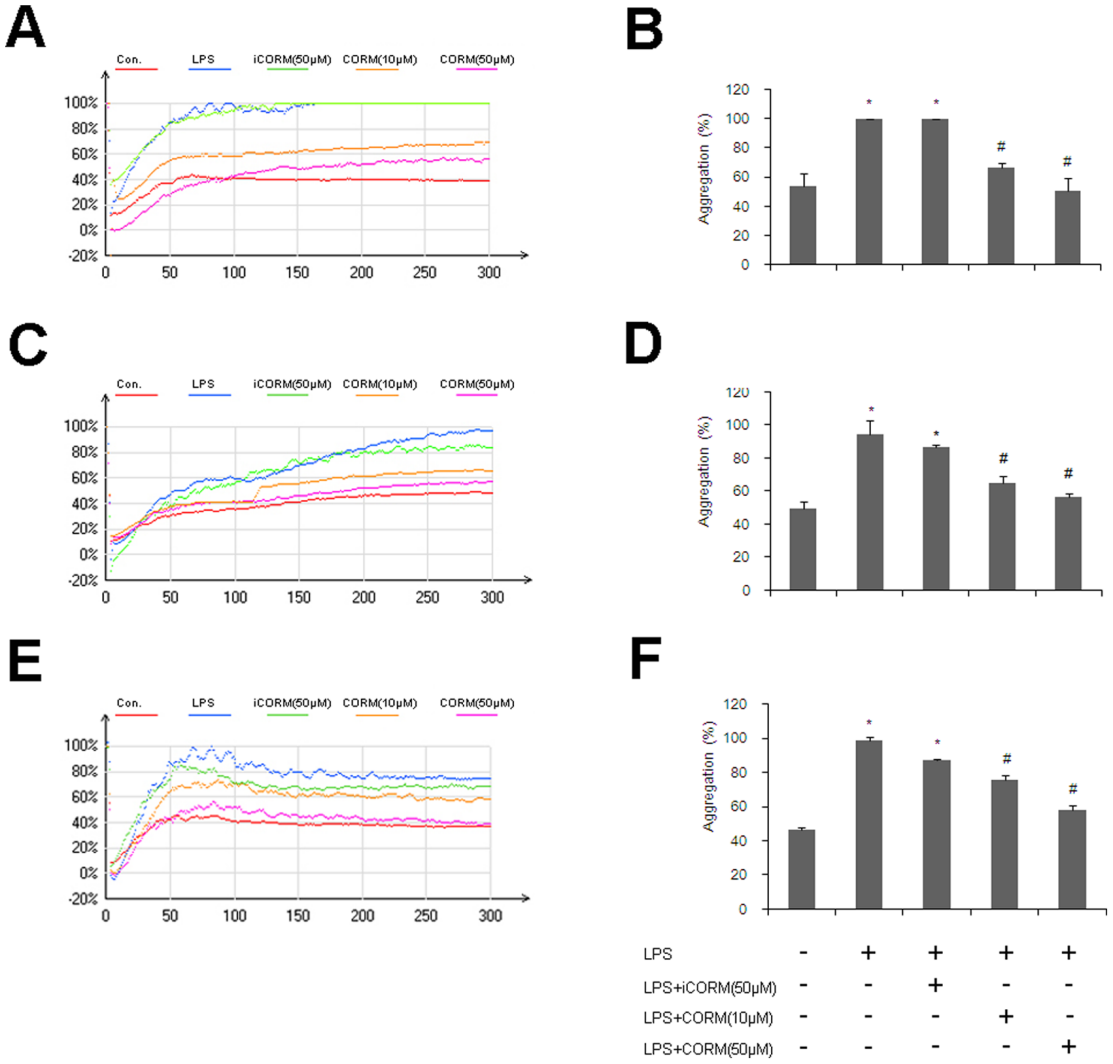
Effects of CORM-2 on aggregation in LPS-stimulated platelets. Platelets were stimulated and intervened as described in Fig. 1. LPS stimulation resulted in a significant increase in platelet aggregation, and treatment of platelets with CORM-2 (10, 50 µM) for 30 min significantly reduced this increase in response to LPS stimulation (A, B). Similar results were also shown in CORM-2 pre-conditioning (C, D) and CORM-2 delayed treatment groups (E, F). The representative images are shown in A, C, and E; the average platelet aggregation ratio of five experiments are shown in B, D and F, respectively. Results are mean ± SE, *P<0.01 as compared to control; #P<0.05 as compared to LPS. Note that LPS-induced platelet aggregation was inhibited by CORM-2 in a dose-dependent manner.

Aggregation function is another important physiological characteristic of platelets, which is one of the most important factors for the role of platelets in hemostasis and thrombosis. LPS- stimulated platelet aggregation was examined using ADP as the inducer. We found that LPS stimulation significantly increased platelet aggregation, and treatment of platelets with CORM-2 for 30 min significantly reduced platelet aggregation in response to LPS stimulation (Figure 2). These results show that LPS played a stimulatory role in inducing platelet aggregation. In addition, these observations showed that this increase was inhibited by administration of CORM-2.

### Effects of CORM-2 on Platelet Granule Secretion in LPS-stimulated Platelets

**Figure 3:**
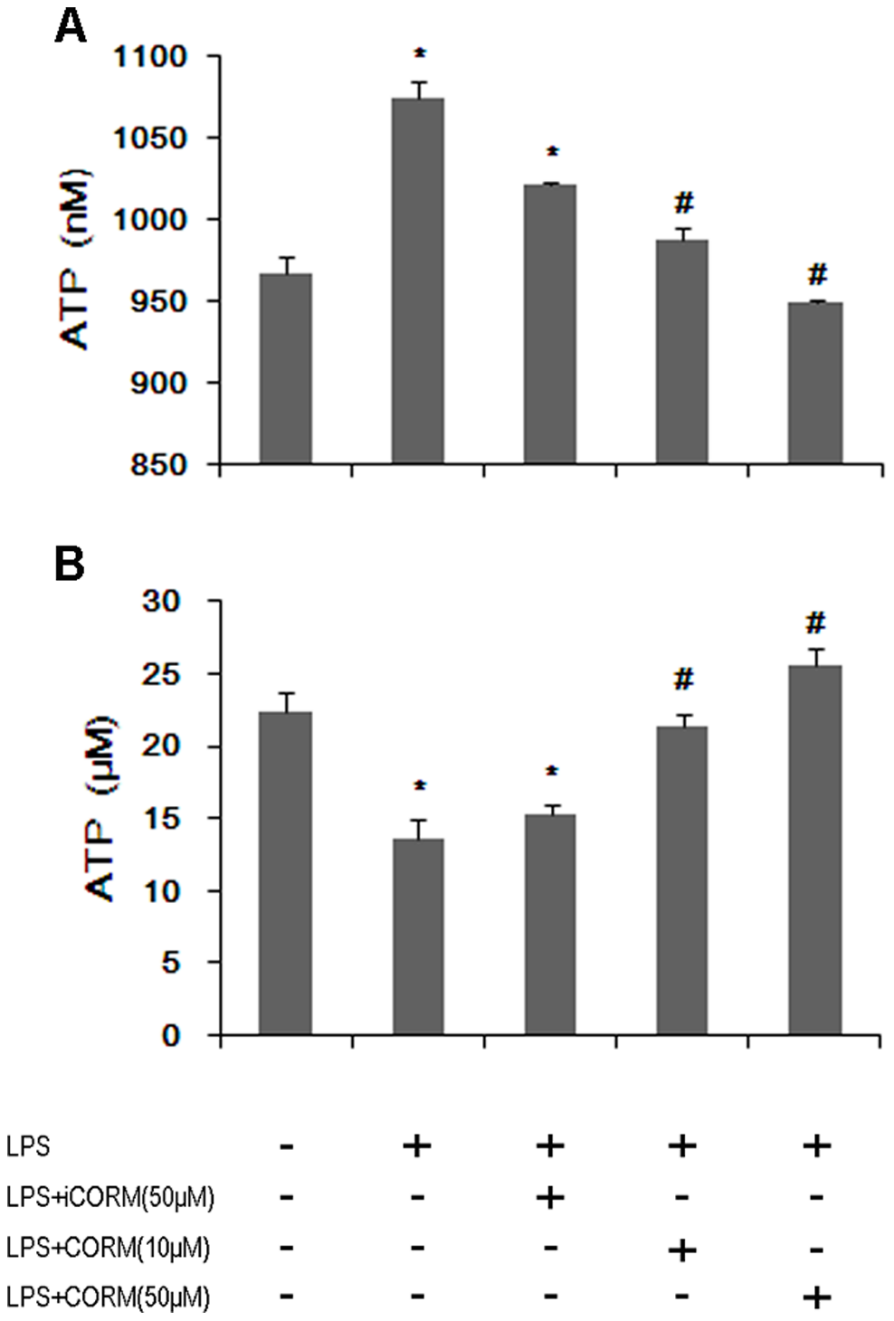
Effects of CORM-2 on granule secretion and intracellular ATP in LPS-stimulated platelets. The platelets were stimulated by LPS and treated with CORM-2 as described in Fig. 1. ATP release in the supernatant (A), and intracellular ATP levels (B) were measured. Results are described as mean ± SE of five experiments, *P<0.01 as compared to control; #P<0.05 as compared to LPS. Note that LPS-induced ATP release was inhibited by CORM-2 in a dose-dependent manner.

Platelet secretion plays an important role in promoting platelet activation induced by low-dose agonists [35]. To determine whether LPS modulated platelet secretion, we examined LPS-induced ATP release in human platelets, which indicated the secretion of dense granules. We found that LPS stimulation significantly increased ATP release and treatment of platelets with CORM-2 for 30 min significantly reduced ATP release in response to LPS stimulation (Figure 3, A). We also observed a change in the levels of intracellular ATP, which were markedly reduced in the LPS group. However, in the CORM groups, the level of intracellular ATP was unchanged (Figure 3, B). Thus, we conclude that LPS stimulates platelet secretion and that exogenous CO alter this phenomenon.

### Effects of CORM-2 on Expression of Platelet Membrane Glycoproteins

**Figure 4:**
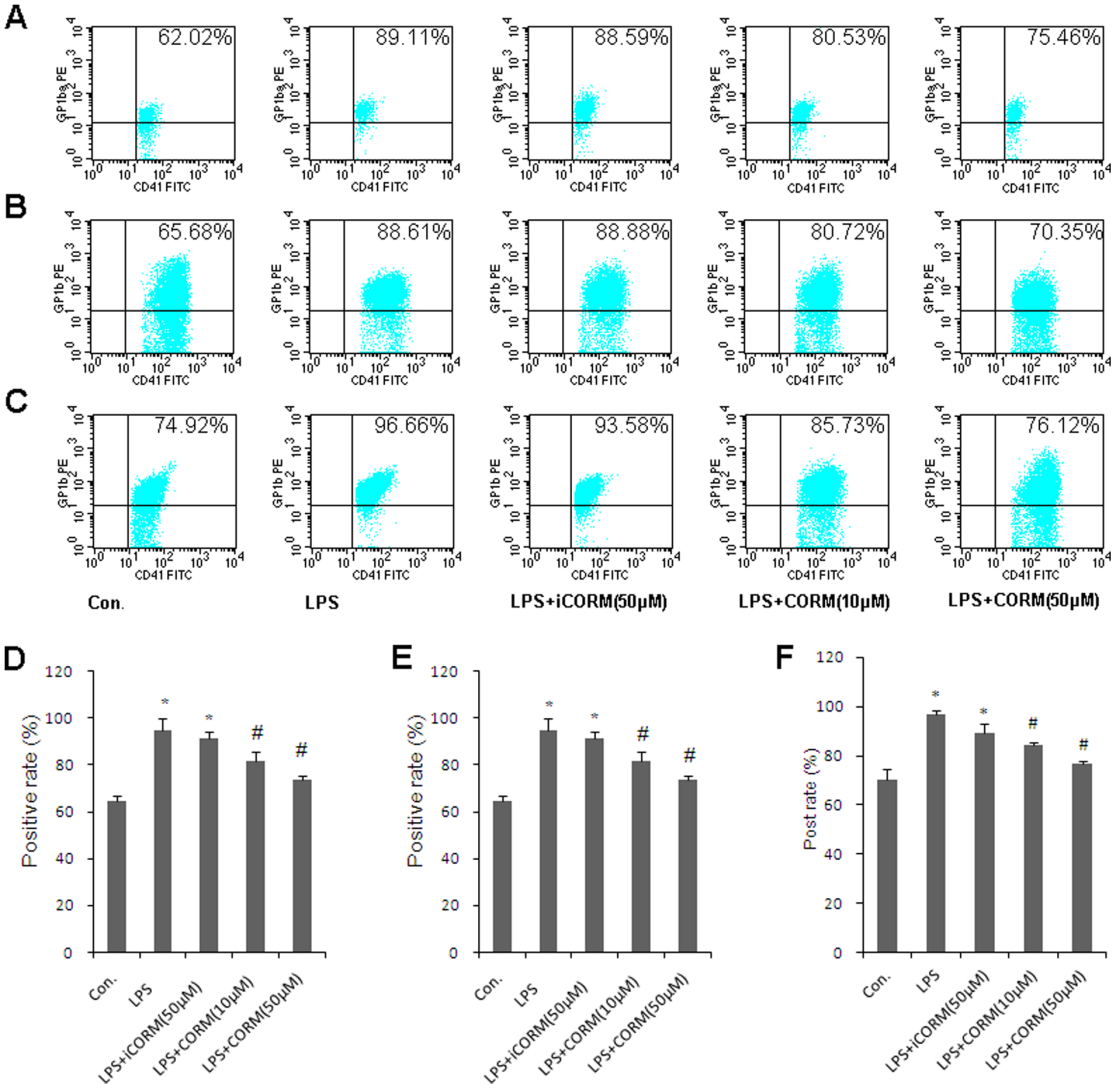
Effects of CORM-2 on expression of platelet membrane glycoprotein GPIbα. The platelets were stimulated by LPS and treated with CORM-2 as described in Fig. 1. Samples were collected and fixed in 1% paraformaldehyde for 15 min at room temperature and incubated with FITC-labeled CD41 (CD41-FITC). GPIbα-PE was added to the samples. All samples were incubated in the dark for 30 min, washed three times and analyzed by flow cytometry. GPIbα expression in LPS-stimulated platelets that were co-incubated with CORM-2 is shown in A. Similar results were also shown in CORM-2 pre-conditioning (B) and CORM-2 delayed treatment groups (C). The average expression rates of GPIbα of five experiments are shown in D, E and F, respectively. Results are described as mean ± SE, *P<0.01 as compared to control; #P<0.05 as compared to LPS. Note that LPS-induced GPIbα expression was down-regulated by CORM-2 in a dose-dependent manner.

**Figure 5:**
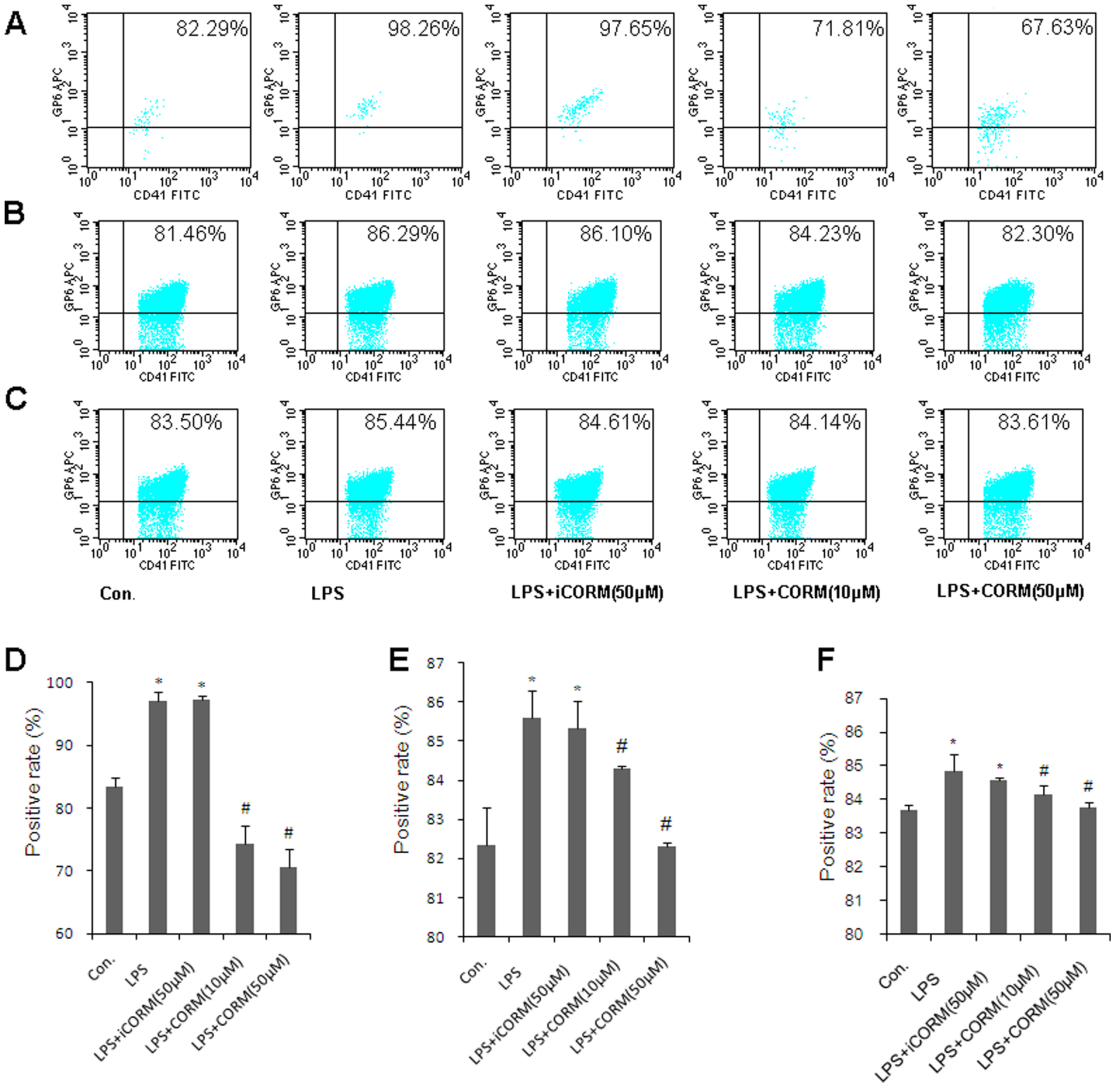
Effects of CORM-2 on expression of platelet membrane glycoprotein GPVI. The platelets were stimulated by LPS and treated with CORM-2 as described in Fig. 1. Samples were collected and fixed in 1% paraformaldehyde for 15 min at room temperature and incubated with FITC-labeled CD41 (CD41-FITC). The GPVI -eFluor 660 was added to the samples. All samples were incubated in the dark for 30 min, washed three times and analyzed by flow cytometry. The GPVI expression in LPS-stimulated platelets that were co-incubated with CORM-2 is shown in A. Similar results were shown in CORM-2 pre-conditioning (B) and CORM-2 delayed treatment groups (C). The average expression rates of GPVI of five experiments are shown in D, E and F, respectively. Results are described as mean ± SE, *P<0.01 as compared to control; #P<0.05 as compared to LPS. Note that LPS-induced GPVI expression was down-regulated by CORM-2 in a dose-dependent manner.

In platelets, it has been difficult to provide direct evidence of a role for receptor-cytoskeletal interactions in the regulation of platelet function. Previous studies suggested that GPIbα and GPVI may regulate platelet function [14], [43]. In the present study, GPIbα and GPVI were measured by flow cytometry to explore the potential mechanism of CORM-2 on the activation of platelets following LPS stimulation. In the LPS group, both GPIbα (Figure 4A) and GPVI (Figure 5A) were significant increased. It is also worth noting that the positive rates of GPIbα (Figure 4D) and GPVI (Figure 5D) were markedly decreased in LPS-stimulated platelets that were co-incubated with CORM-2. Similar results were also shown in CORM-2 pre-conditioning (Figure 4B, E; Figure 5B, E) and CORM-2 delayed treatment groups (Figure 4C, F; Figure 5C, F). Our results suggest that platelet membrane glycoproteins, at least GPIbα and GPVI, were significantly increased in sepsis and that exogenous CO can down-regulate this increase.

### Effects of CORM-2 on Platelet HS1 Tyrosine Phosphorylation

**Figure 6:**
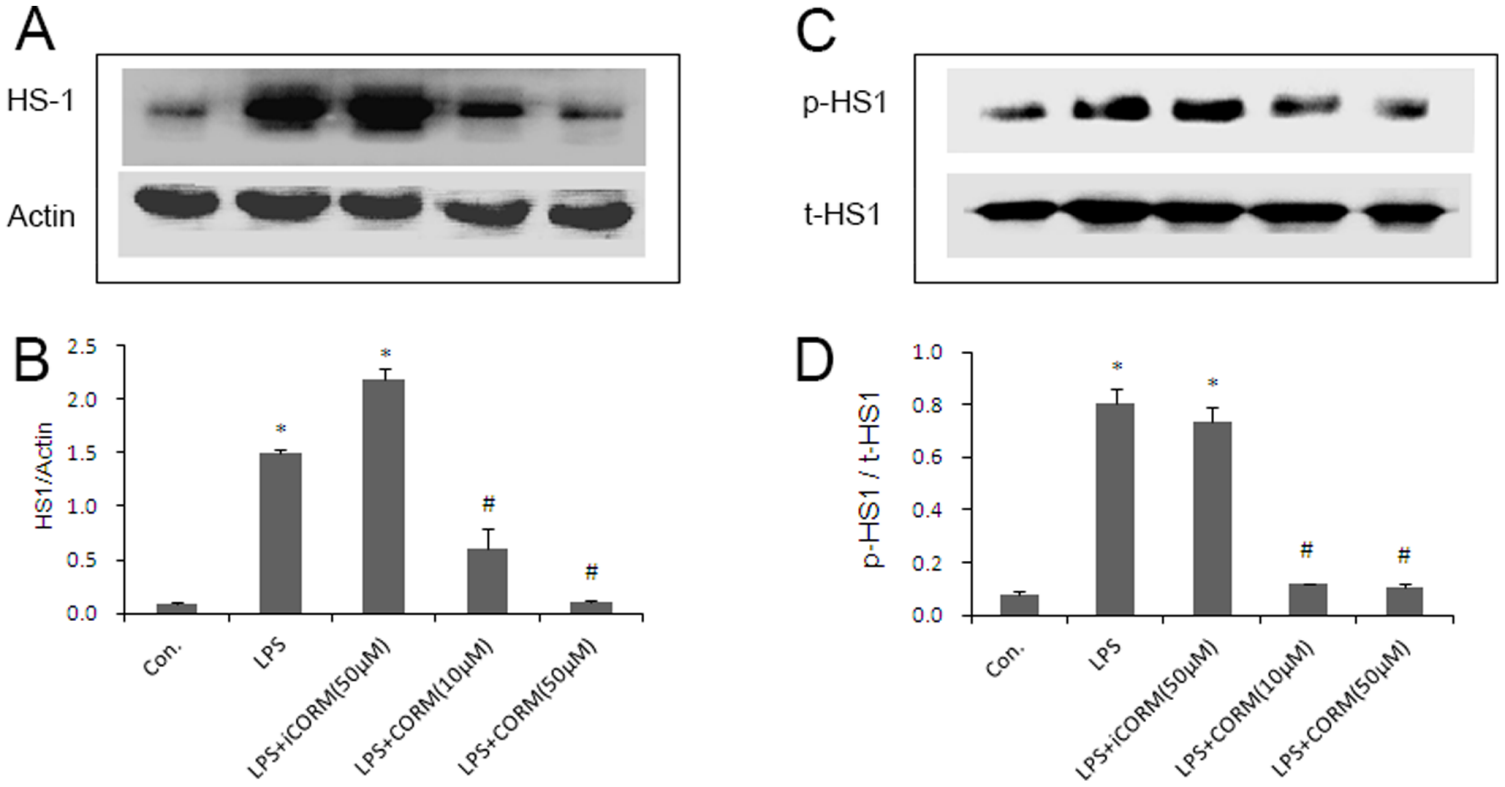
Effects of CORM-2 on platelet HS1 expression and HS1 tyrosine phosphorylation. The platelets were stimulated by LPS and co-incubated with CORM-2 for 30 min. The platelets were lysed in RIPA buffer that contained protease and phosphatase inhibitor cocktails. Platelet HS1 expression and HS1 tyrosine phosphorylation were detected by SDS- polyacrylamide gel electrophoresis and Western blotting. Representative experiments are shown in A and B. The average ratio of HS1/beta-actin and p-HS1/t-HS1 are shown in C and D. Results are described as mean ± SE of three experiments, *P<0.01 as compared to control; #P<0.05 as compared to LPS.

**Figure 7:**
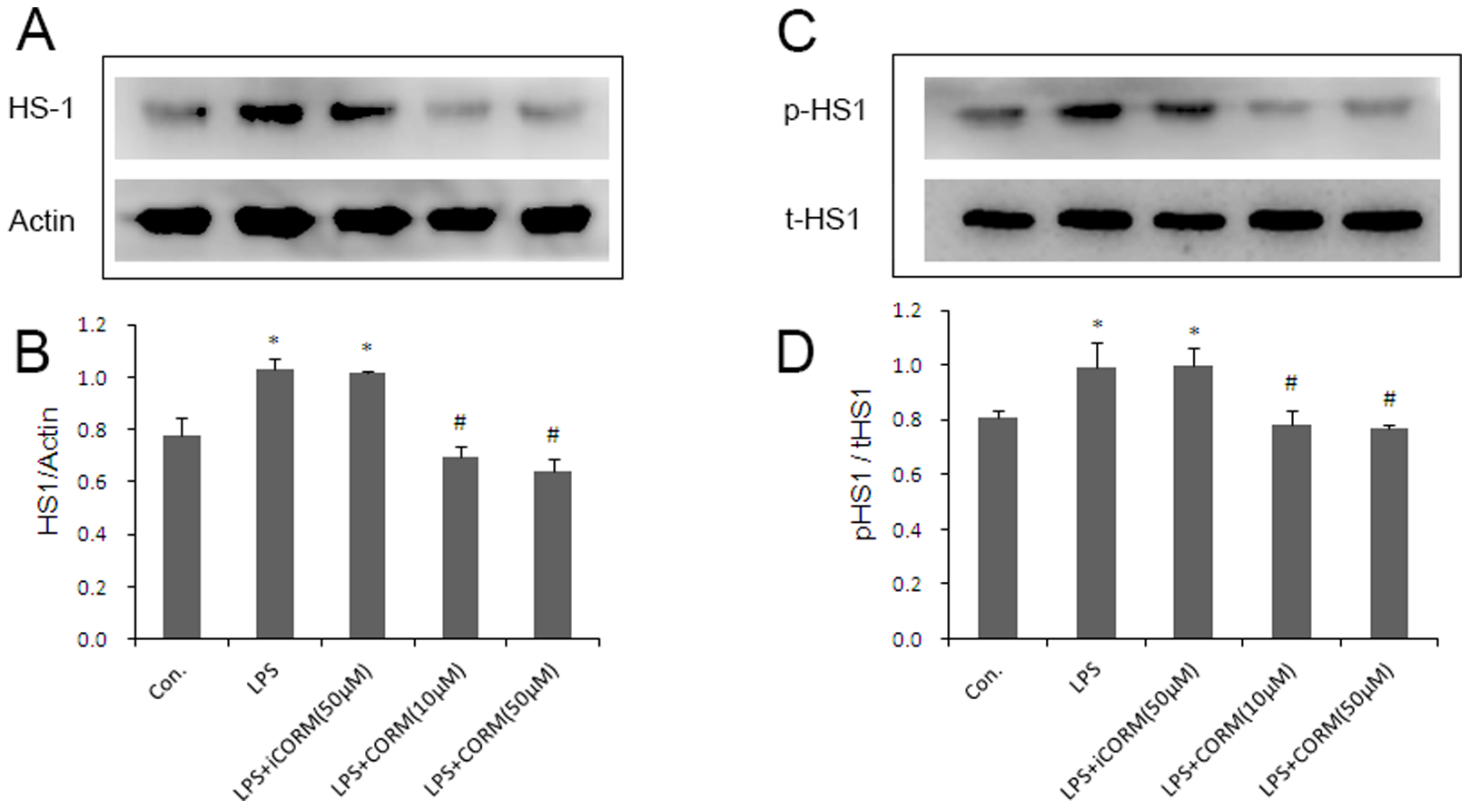
Effect of CORM-2 preconditioning on platelet HS1 expression and HS1 tyrosine phosphorylation. The platelets were pretreated with CORM-2 for 30 min followed by LPS stimulation for an additional 30 min. The platelets were lysed in RIPA supplemented with protease and phosphatase inhibitor cocktails. Platelet HS1 expression and HS1 tyrosine phosphorylation status were detected using SDS-polyacrylamide gel electrophoresis and Western immunoblotting. The representative experiments are shown in A and B. The average ratio of HS1/beta-actin and p-HS1/t-HS1 are shown in C and D. Results are described as mean ± SE of three independent experiments, *P<0.01 as compared to control; #P<0.05 as compared to LPS.

**Figure 8:**
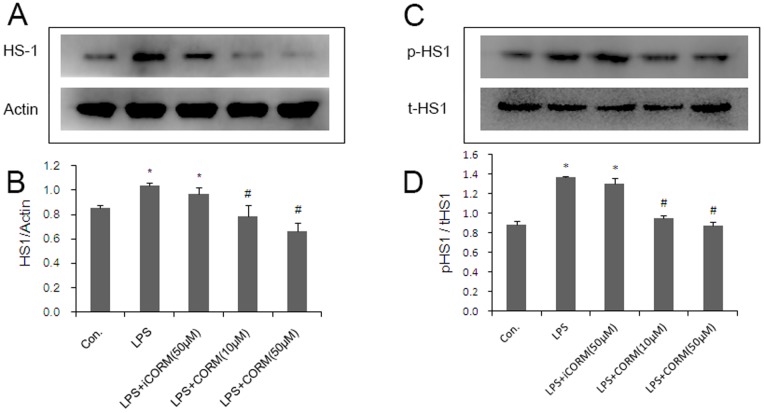
Effects of CORM-2 delayed treatment on platelet HS1 expression and HS1 tyrosine phosphorylation. LPS stimulated platelets (30 min) were treated with CORM-2 for an additional 30 min. Next, the platelets were lysed in RIPA buffer supplemented with protease and phosphatase inhibitor cocktails. Platelet HS1 expression and HS1 tyrosine phosphorylation status were detected by SDS-polyacrylamide gel electrophoresis and Western immunoblotting. The representative experiments are shown in A and B. The average ratio of HS1/beta-actin and p-HS1/t-HS1 are shown in C and D. Results are described as mean ± SE of three independent experiments. *P<0.01 as compared to control; #P<0.05 as compared to LPS.

HS1 plays a key role in platelet functional responses [44] and phosphorylation after activation of the GPVI receptor in platelet stimulation [16]. In the present study, we first examined the activation of HS1 in LPS-stimulated platelets by measuring its phosphorylation status. Our study shows that the amount of HS1 greatly increased when platelets were incubated with LPS alone. We also found that tyrosine-phosphorylated HS1 was significantly increased in LPS groups. However, when the platelets were incubated with LPS and CORM-2, the level of HS1 expression (Figure 6A) and phosphorylated HS1 were downregulated (Figure 6B). Similar results were also shown in CORM-2 pre-conditioning (Figure 7A, B) and CORM-2 delayed treatment groups (Figure 8A, B). These data indicated that LPS stimulated platelet activation may be closely related to HS1 phosphorylation, and that exogenous CO could effectively inhibit this process.

## Discussion

Platelet activation is an important event in the process of activating the coagulation system. Early in sepsis, platelets are activated abnormally. Then, activated platelets adhere to endothelial cells (ECs), which results in EC activation and enhanced expression of EC adhesion molecules, enhanced production of IL-8, and increased leucocyte–EC adhesion [10]. Beside adhesion to ECs, more than 300 proteins and small functional molecules that contribute to the functioning of the vascular wall and circulating immune cells are released. Many of these molecules, including cytokines, chemokines, ADP/ATP and coagulation factors, are preformed and stored in dense bodies or alpha-granules. Other bioactive molecules, including thromboxane, reactive oxygen species (ROS), and IL-1β, are also synthesized by platelets [10], [45]. Furthermore, previous studies have suggested that LPS, plays a fundamental role in sepsis, and could promote platelet aggregation in the presence of plasma [35]. We report herein that CORM-released CO exerts an inhibitory effect against the pathological changes of platelets caused by LPS. For example, exogenous CO effectively inhibited a rise in platelet adhesion, aggregation, and ATP secretion that was induced by LPS. Thus, we propose that CORM-2 contributes to inhibition of platelet activation in sepsis. Moreover, the mechanisms by which platelet activation in sepsis is inhibited remain unknown.

Recent studies have demonstrated various molecular alterations associated with platelets in sepsis. One group of these important molecules are platelet membrane glycoproteins, which have been identified in more than 10 species. The GPIb-V-IX complex, GPVI and protease-activated receptors (PARs) are more closely related to platelet activation. The GPIb complex (GPIb-V-IX) is a heteromeric complex showing continuous expression on the platelet membrane, including four different polypeptide chains, all belonging to the leucine-rich repeat trans-membrane proteins, i.e., GPIbα, GPIbβ, GPV, and GPIX. Several lines of evidence indicate that GPIbα, which is the major receptor of GPIb-V-IX, has a role in many aspects of platelet function, particularly platelet activation by von Willebrand factor (vWF) and thrombin [12], [46], [47]. GPVI is a type I transmembrane glycoprotein of the immune-receptor family, with two extracellular immunoglobulin domains, a mucin domain, a transmembrane domain, and a cytoplasmic tail. Both collagen and laminin are able to signal via GPVI associated with the Fc receptor γ-chain (GPVI/FcR γ), which leads to activation of Syk, and phosphatidylinositol 3-kinase (PI3K). In addition, it is direct interaction between GPVI and GPIbα that promotes a physical and functional co-association of GPVI with the GPIb-IX-V complex [14], [47]–[49].

In this study, we found that expression of both GPIbα and GPVI in LPS-stimulated platelets were significantly up-regulated. This enhanced expression was effectively inhibited by administration of CORM-2. Interestingly, LPS-induced upregulation of membrane glycoproteins were always inhibited no matter which CORM-2 preconditioning or delayed treatment were applied. These results strongly suggest that membrane glycoproteins might be up-regulated by LPS. Administration of exogenous CO reduced the expression of platelet membrane glycoproteins, and inhibited platelet activation in sepsis. Certainly, many other glycoproteins, such as PARs [50], should be further investigated to reveal their variable characteristics during sepsis.

Many results indicate that HS1 is phosphorylated downstream of GPVI activation and depends on the non-receptor Src-family of tyrosine kinases and plays a key role in the functional responses of platelets [16]. HS1 is a 79 kDa intracellular protein kinase substrate that is expressed only in tissues and cells of hematopoietic origin. HS1 is regulated by tyrosine phosphorylation and has Arp2/3-binding capability, F-actin binding-repeats, a coiled coil, is proline rich and displays C-terminal SH3 domains. It has previously been shown that thrombin-induced platelet activation leads to HS1 phosphorylation in a Syk and Src-dependent manner at tyrosine residues 378, 397 and 222 [16], [44], [51]. Furthermore, recent studies have suggested that HS1 is associated with B-Chronic Lymphocytic Leukemia (B-CLL) [52] and systemic lupus erythematosus (SLE) [53]. In this study, we measured HS1 production and phosphorylation levels of platelets that were induced by LPS with or without CORM-2 treatment. There was a significant increase in the levels of HS1 production and phosphorylation following LPS stimulation. These observations suggested that HS1 plays a vital role in LPS-induced platelet activation. However, the phenomenon was significantly inhibited by administration of CORM-2. Similar to membrane glycoproteins, the increase in HS1 expression and phosphorylation in LPS-stimulated platelets are always inhibited irrespective of the CORM-2 preconditioning or delayed treatment. These data showed that CORM-2 exhibits, at least in part, an important role in inhibiting HS1 expression and phosphorylation, and thereby inhibiting abnormal activation of platelets.

In conclusion, the data presented in this study suggests a protective role of CORM-2, which is one of the novel CORMs that is expressed by platelets in sepsis. The potential mechanism of action of the beneficial effect of CORM-2 appears to be mediated by suppressing the expression of platelet membrane glycoproteins, and the production and phosphorylation of HS1, and thereby suppressing abnormal activation of platelets in sepsis. However, the potential benefits of anti-platelet or anti-coagulation strategies in this setting should be validated by further studies.
